# Edge structure influences infants’ and toddlers’ engagement with natural scenes

**DOI:** 10.3758/s13414-026-03357-x

**Published:** 2026-10-28

**Authors:** Brianna K. Hunter, Kaitlyn Wong, Lisa M. Oakes

**Affiliations:** 1 Center for Mind and Brain, University of California at Davis, 267 Cousteau Pl, Davis, CA 95695 USA; 2 Department of Psychology, University of California at Davis, Davis, CA USA

**Keywords:** Development, Scene Perception, Attention

## Abstract

**Abstract:**

Low-level features, or basic visual properties like color, luminance, and orientation, guide visual attention across the lifespan. We evaluated how variations in one low-level feature, edge structure, related to visual attention to natural scenes (i.e., photographs of places such as kitchens and landscapes) across the first two postnatal years of life. Specifically, we asked how the proportion of cardinal edges (i.e., horizontal and vertical) as well as the edge quantity within scenes related to infants’ (*N* = 107, 5 to 12 months; 63 girls and 44 boys, 39.25% were White and not Hispanic, and the remaining infants were Asian, Black, Multiracial, and/or Hispanic) and toddlers’ (*N* = 79, 13 to 21 months; 39 girls, 39 boys, 1 not reported, 45.57% were White and not Hispanic, and the remaining toddlers were Asian, Black, Multiracial, and/or Hispanic) visual attention to those scenes. We found that infants looked longer at scenes with more edges and decreased their looking to scenes with a higher proportion of cardinal edges. In contrast, toddlers looked longer at scenes with a moderate proportion of cardinal edges, and their looking was unrelated to edge quantity. Within both samples, we found that the role of these edge properties changed with age. Therefore, there appear to be developmental changes from infancy to toddlerhood in the role of edge structure on children’s attention to natural scenes.

**Open practices statement:**

Materials that support the findings of this study are openly available on OSF (https://osf.io/zdghu). We provide fixation data for both experiments, analysis code, and experiment stimuli. Experiments were not preregistered.

**Supplementary Information:**

The online version contains supplementary material available at https://doi.org/10.3758/s13414-026-03357-x.

Low-level perceptual features such as color, luminance, and orientation are fundamental building blocks within a hierarchical visual system. They support progressively higher-level visual representations, ranging from simple shapes to complex scenes (Epstein et al., 1999; Torralba & Oliva, 2003). These low-level perceptual features not only shape the development of visual cortex and perceptual abilities during infancy (Hirsch & Spinelli, 1970) but also guide attentional processes (Colombo, 2001; Helo et al., 2017; Henderson & Hayes, 2017; B. K. Hunter et al., 2025a, b; Pomaranski et al., 2021). The role of low-level features in the guidance of attention is particularly pronounced in early development. The development of visual attention has classically been described as a shift across the first postnatal year, with early looking behaviors driven by these low-level features, and attention becoming increasingly guided by higher-order factors such as meaning and social relevance (Colombo, 2001). However, the vast majority of this research has examined infants’ attention to relatively simple stimuli, and therefore limited data exist on infants’ sensitivity to natural scene features (Newman et al., 2025). Addressing this gap was a primary goal of the present work.

Importantly, although several studies have found that younger infants are more likely to fixate on regions of scenes that are highly physically salient, whereas older infants tend to fixate on regions containing semantically meaningful or social information (e.g., Frank et al., 2009; Oakes et al., 2024), low-level features continue to contribute to attentional guidance throughout infancy (Hunter et al., 2024; Hunter et al., 2025a, b; Oakes et al., 2024), childhood (Açık et al., 2010; Helo et al., 2017), and into adulthood (Henderson & Hayes, 2017). Therefore, changes in attention across the first postnatal year do not reflect a loss of sensitivity to low-level features over development; rather, they reflect changes in the relative weighting of low-level and higher-level influences across development. Moreover, sensitivity to some features of natural scenes actually increases across infancy (Newman et al., 2025). Together, this work suggests that developmental changes in visual attention may reflect the dynamically changing contributions of multiple sources of visual information.

In the current study, we examined the role of one low-level feature, edge structure, in infants’ and toddlers’ attention. We focus on edges, defined as the abrupt intensity changes in the visual field, due to their unique functional role in visual processing. Edges delineate object boundaries, segment surfaces, and provide a foundation upon which higher-level scene meaning can be built. McAdams et al. (2025) found that infants’ visual preferences for scenes were determined, in part, by the edge structure in those scenes. Furthermore, edge orientation is encoded in early visual cortex (Jehee et al., 2012; Mazer et al., 2002), and representations in this region strongly influence gaze during the first year of life, particularly during early infancy (Hunter et al., 2025a, 2025b). We therefore analyzed how both the *quality* of the edges (i.e., the proportion of edges along the horizontal and vertical axes) and the *quantity* of edges in complex natural scenes (e.g., photographs of real-world environments such as kitchens and landscapes) relate to attention to those scenes in the first and second postnatal years.

## The significance of cardinal orientations for visual processing

Typical views of the real world over-represent horizontal and vertical contours (i.e., cardinal orientations; Barlow, 1959; Coppola et al., 1998); this preponderance of cardinal orientations is evident from egocentric views of infants as early as 3 weeks of age and is stable by around 25 weeks (Petroff et al., 2025). A rich body of evidence suggests that exposure to specific edge orientations in the environment plays a foundational role in the development of visual cortex. Classic studies showed that kittens raised in environments with only horizontal or vertical lines became behaviorally “blind” to the orientation orthogonal to the ones they had experienced, and their orientation-selective neurons were disproportionately tuned to the experienced direction (Blakemore & Cooper, 1970; Hirsch & Spinelli, 1970). Even following years of normal visual experience, these orientation biases in kittens persisted, indicating long-lasting effects of orientation experience on the tuning of early visual cortex (Blasdel et al., 1977).

In humans, it is theorized that this naturally occurring statistical regularity in the visual environment enhances perceptual sensitivity to cardinal orientations, facilitating more efficient visual processing in early visual cortex (Barlow, 1959). Indeed, when presented with lines of varying orientations, both adults and infants show enhanced perception of vertical and horizontal stimuli compared with oblique orientations, a phenomenon widely known as the *oblique effect* (Gwiazda et al., 1978; Jastrow, 1892; Sokol et al., 1987). Together, these findings highlight the important role of edge orientation in the development of visual cortex and perceptual abilities.

Despite the foundational role of edge orientations in the development of visual and perceptual systems, limited work has examined how the developing attention system in humans responds to this information. B. K. Hunter and colleagues (2024) evaluated which specific features within a quantitative model of low-level stimulus salience best predicted infants’ attention during natural scene viewing. They found that fixations were most strongly related to the model’s “orientation” feature, referring to the distinctness of edges or contours at specific angles. Furthermore, McAdams and colleagues (2025) found that when infants were presented with images of two building facades side by side, they looked longer at facades with greater randomness in their edge orientations. Together, these results provide initial evidence that orientation plays a significant role in guiding infants’ visual attention to complex scenes. However, the contribution of specific, ecologically relevant orientations (e.g., cardinal orientations) remains unknown.

In adults, it appears that the attentional system is indeed sensitive to specific orientations. Hansen and Essock (2004) evaluated adults’ attentional sensitivity to cardinal and oblique gratings overlaid on images of natural scenes and found that participants were most effective at detecting oblique orientations. The authors proposed that because the visual system is optimized for processing cardinal orientations, attention to these cardinal gratings was suppressed. In contrast, the oblique gratings overlaid on scenes were less expected and thus more salient to the attention system.

Predicting how the infant and toddler attention system will respond to cardinal and oblique edges is complicated. For decades, researchers have explored attentional preferences in infancy to relatively novel versus relatively familiar stimuli (Fagan, 1970; Houston‐Price & Nakai, 2004; Hunter et al., 1983; Rose et al., 1982). Because Petroff et al. (2025) found that cardinal edges are more common in infants’ egocentric views compared with oblique edges, it is possible that infants will be most engaged with scenes containing relatively less cardinal edges because they are more novel. However, some studies have shown that infants instead look longer at highly familiar stimuli, such as images of their own mother compared with a stranger (e.g., Barrera & Maurer, 1981) or images of own-race (or familiar-race) individuals compared with other- (or unfamiliar-race) individuals (Kelly et al., 2005). In this case, infants may look longer at scenes with relatively more cardinal edges because they are more similar to their daily views. In either case, we predict that infants’ and toddlers’ looking will be systematically related to the proportion of cardinal edges. Such findings would indicate that the structure of infants’ everyday visual experience shapes how they allocate attention to environments with common versus uncommon edge orientations.

## Developmental changes in sensitivity to edge quantity

In addition to the role of specific edge orientations, the *quantity* of edges in scenes may be related to attention during early development. We hypothesized that edge quantity, defined as the number of pixels in an image that are part of an edge, would relate to infants’ attention for two primary reasons. First, as with the proportion of cardinal edges, variations in edge quantity may reflect variations in familiarity. In an analysis of data from video cameras mounted on the foreheads of 3-week-old to 13-week-old infants, Anderson et al. (2024) found that infants’ visual environments contained significantly fewer edges compared with those of adults. To be clear, young infants’ egocentric scenes are not lower in edge quantity solely because infants *select* views of the world that have fewer edges; rather, infants’ views of the world are constrained by their physical size and motor abilities (Smith et al., 2018). Thus, as they explore their environment, infants’ egocentric views have fewer edges because the infants are lower to the ground, their arms are relatively short (and thus the objects they hold are closer to their face), and other physical and motor features. Therefore, scenes with more edges may be relatively novel compared with the views they can create of the world. As described previously, infants indeed demonstrate visual preferences based on the relative familiarity versus novelty of stimuli (Fagan, 1970; Houston‐Price & Nakai, 2004; Hunter et al., 1983; Rose et al., 1982).

A second reason that edge quantity may relate to attention is because variations in edge quantity may reflect variations in perceived complexity. Research indicates that infants’ looking durations when viewing relatively simple stimuli (e.g., black-and-white checkerboards) are influenced by the “complexity” of those stimuli (e.g., 24 × 24 vs. 2 × 2 checks; Cohen et al., 1975). In these simple displays, complexity is defined in terms of the number of checks. In more cluttered scenes, complexity may instead be defined by the number of edges, or edge density (Rosenholtz et al., 2007). Therefore, when viewing more cluttered scenes, infants’ looking duration may be related to edge quantity.

As with the proportion of cardinal edges, it is not immediately clear whether infants will look longer at scenes with higher or lower edge quantities. Infants may look longer at scenes with more edges because they are more novel or instead scenes with fewer edges because they are more familiar based on infants’ typical view of the world. Song et al. (2021), for example, found that adults viewing paintings sometimes showed gaze preferences for more familiar images and sometimes showed gaze preferences for more novel images. Specifically, whereas adults showed novelty preferences for complex landscapes and abstract paintings, they showed familiarity preferences for simple portraits. Moreover, a large literature has led to the conclusion that infants prefer more complex stimuli (e.g., Cohen et al., 1975; Fantz & Fagan, 1975; Wilcox, 1969). In this case, we predict that infants will look longer at scenes with more edges. Although the direction of this effect is not certain, any systematic relation between edge quantity and infant looking will reveal how infants’ attention is influenced by the visual structure of the environments they encounter.

Much less is known about the factors influencing looking behaviors during toddlerhood. One reason for this gap is that research on infant scene viewing has found that attention becomes increasingly systematic and “adult-like” across the first postnatal year (Pomaranski et al., 2021). Specifically, older infants demonstrate more consistent fixation patterns compared with younger infants, which increasingly resemble those of adults, suggesting linear progression over time. Supporting this idea, Helo and colleagues (2017) evaluated 24-month-old toddlers’ attention to natural scenes and found that low-level features had a stronger role in determining where toddlers looked compared with adults. This suggests that, despite similarities between adult and older infants’ fixations observed by Pomaranski et al. (2021), the relative weighting of low-level features continues to shift well into the second year of life. However, other work shows that toddlers’ attention to natural scenes shifts during this period as the prioritization of different types of higher-level information changes. For example, Dalrymple and colleagues (2019) found that at 18 months, toddlers’ attention was best defined by their increased interest in faces, but at 30 months, their attention was best defined by their increased interest in things that are interacted with by others (e.g., gazed at, touched), reflecting nuanced changes in the higher-order factors that guide attention. Together, this work demonstrates that the features guiding attention continue to change throughout the second and third postnatal years, underscoring the importance of studying attention development beyond the first 12 months of postnatal life. Moreover, although low-level features appear to continue to influence toddlers’ attention to natural scenes (Helo et al., 2017), given the significant development of the visual system in the first 12 months (Atkinson & Braddick, 2020; Kellman & Arterberry, 2000), it is likely that there are significant changes from infancy into toddlerhood in the role of specific features on attention guidance.

Overall, given the large changes in the visual system during infancy, we expected that the influence of edge structure on attention would change during the first year. This prediction is based on the classic view of visual attention development, which emphasizes a decreasing role of low-level features as attention becomes increasingly guided by higher-order factors (Colombo, 2001). However, we expected that edge structure would also have a significant effect on attention across the entire age range tested. Both infants and adults demonstrate enhanced perceptual sensitivity to cardinal orientations (Gwiazda et al., 1978; Jastrow, 1892; Sokol et al., 1987), suggesting that edge structure may indeed strongly influence attentional processes during both infancy and toddlerhood. However, given the limited research on toddlers, our analyses of toddlers were largely exploratory and aimed to determine if and how low-level properties related to edges and orientation shaped attention in the second postnatal year.

## The present study

The present study examined the role of various edge properties of natural scenes on infants’ and toddlers’ looking behaviors. Specifically, we asked in two experiments how the attributes of the proportion of edge orientations on the cardinal axis and edge quantity influence how long infants and toddlers maintained attention to complex natural scenes. Experiment 1 examined looking behaviors from infants between the ages of 5 and 12 months, whereas Experiment 2 examined looking behaviors from toddlers between the ages of 13 and 21 months.

Our primary predictions for our infant sample were based on findings from the literature. We expected to find that infants’ looking would vary with the proportion of cardinal orientations and number of edges within the scenes, but the direction of these effects were unclear. Longer looking to scenes with a smaller proportion of cardinal edges and more edges overall would be consistent with infants preferring to look at scenes containing relatively novel visual structure (Anderson et al., 2024; Coppola et al., 1998; Petroff et al., 2025), whereas longer looking to scenes with greater proportion of cardinal edges or fewer edges overall would be consistent with infants preferring to look at more familiar structure. Moreover, scenes with more edges are more complex (Rosenholtz et al., 2007), and complexity may independently influence engagement with scenes, as it does for simpler visual displays (e.g., Cohen et al., 1975; Fantz & Fagan, 1975; Wilcox, 1969). Thus, both novelty and complexity provide potential explanations for greater looking to scenes with more edges, whereas a preference for familiarity would predict the opposite pattern.

Our predictions for toddlers’ interest in these scenes were more speculative, as less work has established the basis of longer looking in this age range. However, we predicted the same general patterns for the toddler sample as for the infant sample, given findings that 9-month-old to 41-month-old children show visual preferences for shapes based on their complexity (Munsinger & Weir, 1967).

We also predicted that the effect of these edge properties would change across this developmental period. In particular, we expected that for the infant sample, edge properties would have stronger effects at earlier versus later timepoints, based on evidence that low-level features have a stronger influence on attentional guidance in younger infants (Colombo, 2001). Examination of the toddler sample will provide insight into whether the effect of edge structure continues to develop beyond the first year.

## Method

Data from Experiment 1 have been previously published (Hunter et al., 2025a, b), but the measures and analyses reported here are original and have not been previously published. Experiment 2 (the toddler sample) was also collected as part of a larger study and the session included multiple eye-tracking tasks and free-play tasks; only the scene viewing task will be reported here. Neither of the experiments was preregistered.

### Participants

Before engaging in analyses, we determined the appropriate sample size for our planned analyses. Thus, sample size was determined a priori by simulating repeated-measures data using population values based on the smallest effect size of interest (Cohen’s *f* = 0.1; Anvari & Lakens, 2021)*.* Our simulations indicated that a final sample of 40 participants per age group would achieve a power of 0.8*.* Given the samples available, we therefore determined we had adequate power to conduct these analyses.

Families were recruited from two sources. For Experiment 1 (infants), we used birth records obtained by the California State Office of Vital Records to send parents information about our research in general, and parents interested in taking part in the research contacted us by phone, email, webform, or returning a postage-paid card with their contact information. When their child reached an eligible age for the study, parents were contacted and scheduled for an in-lab appointment. For Experiment 2 (toddlers), eligible families were also identified from electronic medical records from UC Davis Health and sent an invitation to the study via text or email using a secure messaging system. Parents who were interested responded, completed a webform and were scheduled for an in-lab appointment if their child was eligible. Families were compensated with a certificate of completion and a small toy (infants, Experiment 1) or a $50 gift card (toddlers, Experiment 2) at the end of each visit.

#### Experiment 1

The final sample included 107 infants tested in Davis, CA, between April 18, 2023 and July 25, 2024. Infants were recruited from the suburban and urban regions surrounding the Sacramento Valley in two age groups: 5-months to 7-months and 10-months to 12-months (see Table 1 for age distributions). Note that the infants included in the final sample differ slightly from those reported in B. K. Hunter et al., (2025a, b), due to the analyses in that publication requiring different data cleaning and exclusionary criteria. All infants were full term, typically developing, and reflected diverse backgrounds consistent with the local demographics. Sixty-three were girls and 44 were boys. Fifty-nine were White (17 Hispanic), 11 were Asian or Asian American (zero Hispanic), three were Black or African American (two Hispanic), 30 were multiracial or other (nine Hispanic), and four had no race reported (four Hispanic). Families were primarily middle-class; 103 mothers had graduated high school, and 82 mothers had completed at least a 4-year degree. Full demographics can be found online (https://osf.io/zdghu).

**Table 1:** Age distribution and demographics for each age group

	*N*	Mean age (*SD*)	Age range
Experiment 1: Infants			
5–7 months	53	183.09 days (17.88)	154–213 days
10–12 months	54	341.93 days (14.57)	307–366 days
Experiment 2: Toddlers			
13–15 months	38	434.74 days (12.27)	412–458 days
19–21 months	41	610.98 days (11.91)	594–638 days

We tested 16 additional infants but excluded them from final analyses due to fussiness (*n* = 8), equipment error (*n* = 2), parental interference (*n* = 2), failure to successfully calibrate (*n* = 3), or not contributing sufficient data (*n* = 1; see Fixation Definitions and Inclusion Criteria).

#### Experiment 2

The final sample included 79 toddlers tested in Davis, CA, between May 25, 2024 and September 13, 2025. Toddlers were recruited from the same population as the infant sample and tested at two age groups: 13 months to 15 months or 19 months to 21 months (see Table 1 for age distributions). Toddlers were typically developing and reflected diverse backgrounds. Thirty-nine were girls and 39 were boys (one did not have sex reported). Forty-six were White (nine Hispanic, one ethnicity not reported), nine were Asian or Asian American (two Hispanic), two were Black or African American (zero Hispanic), 20 were multiracial or other (11 Hispanic), one was American Indian or Alaskan Native (one Hispanic) and one had no race reported (one Hispanic). Families were primarily middle-class; all mothers had graduated high school, and 72 mothers had completed at least a 4-year degree. Full demographics can be found online (https://osf.io/zdghu).

Seven of these toddlers were also a part of the 5–7-month-old infant sample. However, the sessions were spaced far apart (*M* = 10.77 months, *SD* = 3.48, range: 7.14–15.65 months), the overall testing procedures differed (see Procedure), and only a subset of scenes was randomly selected from a larger pool during each visit. Therefore, there was not complete overlap in the scenes presented, and for those that did repeat, we do not expect the toddlers to have remembered them. Nonetheless, we conducted secondary analyses restricted to scenes that were viewed in the lab for the first time. The findings of these analyses mirror those reported here and are reported in the Supplementary Materials.

We tested 17 additional toddlers but excluded them from final analyses due to fussiness (*n* = 1), failure to successfully calibrate (*n* = 7), or ineligibility due to health or vision concerns (e.g., familial risk for colorblindness; *n* = 9).

### Stimuli

**Fig. 1:**
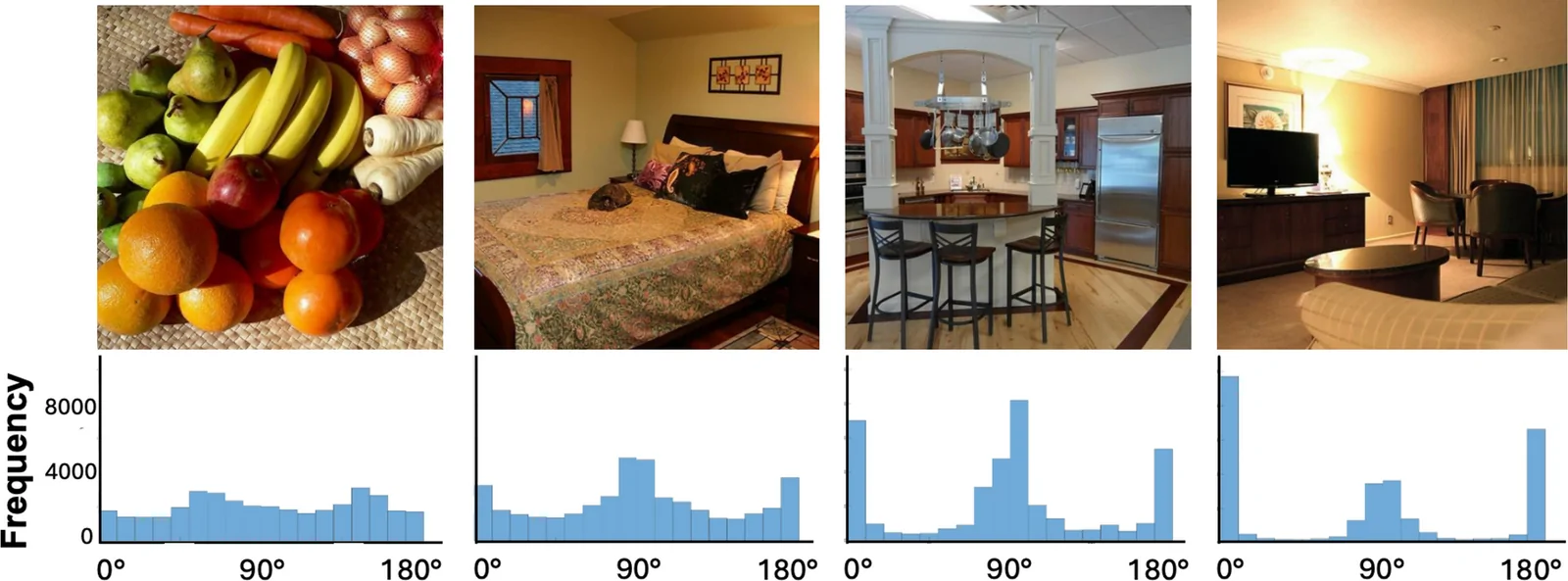
Examples of stimuli and associated orientation histograms. Orientation distributions are collapsed to the range 0°–180° for visualization, such that opposite orientations (e.g., 30° and 210°) are treated as equivalent. See the full stimulus set and associated orientation histograms at https://osf.io/zdghu

For both experiments, the stimuli were 78 scenes from the COCO image set (see Fig. 1; Lin et al., 2015). The image content included a wide range of objects and locations expected to be generally familiar to participants, such as living rooms, buildings, animals, landscapes, and parks (61 images depicted indoor spaces and 17 were outdoor; 25 contained people or animals and 53 did not).^1^ Images were 1,000 × 1,000 pixels (approximately 26˚ × 26˚ of visual angle at a viewing distance of 60 cm) and were centered on the monitor (53 × 30 cm, 1,920 × 1,080 pixels). The remainder of the monitor display was gray for Experiment 1 (infants) and white for Experiment 2 (toddlers).

### Apparatus

For both experiments, fixations were captured using an SR-Research EyeLink1000 Plus eye tracker with a sampling rate of 1000 Hz. Infants’ eye movements (Experiment 1) were recorded using a 25-mm lens and 940-nm illuminator, and toddlers’ eye movements (Experiment 2) were recorded using a 16-mm lens and 890-nm illuminator. The eye tracker was attached to a 24-in. ASUS vg248 monitor display, and both could be moved and adjusted using a moveable arm. We programmed the experiment in Experiment Builder, the software provided by SR-Research for this purpose. This program controlled stimulus presentation (i.e., which stimulus, for how long) and collected eye gaze information during the session. In addition, an Anivia W8 1080p webcam was placed on the top of the monitor to display the participant’s behavior to the experimenter during the eye-tracking session.

### Procedure

The following protocols were reviewed and approved by the UC Davis Institutional Review Board. We obtained informed consent either over the phone or in person from a parent or guardian of each participant prior to the study.

Participants sat approximately 60 cm from the monitor in a caregiver’s lap or a high chair with their parent or guardian nearby. Parents wore felt-covered glasses to obstruct their view of the experimental stimuli and to ensure their looking behavior did not influence their child’s looking behavior. Each session began with a standard 5-point calibration procedure. A looming circle appeared in a target location, starting with the center. Once a fixation was detected at one calibration location, the looming circle moved to the other calibration points. After this calibration procedure, a validation procedure immediately followed, in which the same looming circle appeared at each of the previous locations to validate the accuracy of the calibration procedure. Calibration was repeated if calibration accuracy was poor (e.g., average systematic error > 2° overall or a fixation appeared in an obvious outlier position). The average calibration for participants included in the final analysis was good for both the infant (*M* = 0.82°, *SD* = 0.53°) and toddler (*M* = 0.66°, *SD* = 0.27°) samples. Following calibration and validation, the presentation of experimental trials varied depending on Experiment.

#### Experiment 1 (infant sample)

Experimental trials began immediately after calibration. Each trial was preceded by a small animated cartoon character presented at the center of the monitor, paired with attention-getting sounds (e.g., bells, rattles), to orient the infant to the center of the display. Once the eye tracker detected a fixation of 500 ms to the attention-getting stimulus, a single stimulus image was presented on the monitor for 5 s. If the infant did not fixate on the attention getter, the experimenter had the option to manually progress to the stimulus presentation, but because the infant was not fixating at the center of the screen at the start of the trial, we did not include those trials in our primary analyses (see Fixation Definitions and Inclusion Criteria and related analyses in the Supplementary Materials). The stimulus image was accompanied by classical music to keep the infant interested in the task. On each trial, a different random scene was selected from the stimulus set. This procedure continued until either 1) the infant viewed 50 of the 78 possible scenes, 2) the participant became fussy and refused to continue looking at the monitor, or 3) the eye tracker lost track of the infant’s eye. Thus each infant received a slightly different collection of stimuli in a different random order. Experimental sessions lasted approximately 5 min.

#### Experiment 2 (toddler sample)

The scene stimuli were presented in a session in which scene trials were interleaved with trials for a visual short-term memory task. These nonscene trials consisted of presentations of arrays containing various colored circles and were included for purposes outside the scope of the current study and will not be analyzed here. Each experimental trial was preceded by a flashing fixation cross to orient the toddler to look at the center of the screen. Once the eye tracker detected a fixation of 500 ms to the attention getter, a single stimulus image was presented for 6 s. On each trial, a different random scene was selected from the stimulus set. If the toddler did not fixate on the attention getter, the experimenter did not have the option to manually progress to the stimulus presentation. In such cases, the experimenters used toys or hand gestures to reengage the child; if unsuccessful, the experimenters terminated the experiment, retaining the data from the trials that were completed. The stimulus image was also accompanied by classical music to maintain task engagement. After every five scenes, the eye tracker performed a drift check to confirm that calibration accuracy had not declined. If necessary, the experimenter could recalibrate the eye tracker but this was not required for any subjects. This procedure continued until either 1) the toddler viewed 51 scenes, or 2) they became fussy and refused to continue looking at the monitor. The entire experimental sessions (including scene trials and visual short-term memory trials) lasted approximately 20 min.

### Data processing

#### Fixation definitions and inclusion criteria

Fixations for both experiments were defined using SR-Research’s default fixation detection parameters in Data Viewer; all fixations had a minimum duration of 80 ms and were defined as periods of stable gaze between velocity-defined eye movements (30°/s velocity; 8,000°/s^2^ acceleration). For Experiment 1, we began with 4,378 trials before applying any exclusionary criteria. We excluded trials in our analyses if the infant did not look at the attention getter prior to the trial (i.e., the experimenter manually progressed the experiment; *n* = 248 trials), if the infant made only one fixation on the scene (*n* = 92 trials), or if the infant only looked at the gray portion surrounding the scene that did not have any scene content (*n* = 18 trials).^2^ After imposing these criteria, one infant was excluded from analyses due to having no usable data (see Participants section for Experiment 1). Thus our final analyses for Experiment 1 were conducted on 4,020 trials across 107 infants, as they viewed 78 unique scenes.

For Experiment 2, we began with 2,023 trials. The experimenter did not have the option to manually progress the experiment without the toddler first looking at the attention getter (i.e., all trials began with the toddler looking at the fixation cross) so that exclusionary criterion was not applied. We excluded 21 trials because the toddler only made one fixation, and three trials because the toddler only looked at the white portion of the screen without scene content. No toddlers were excluded after the trial exclusions were imposed. Thus our final analyses for Experiment 2 were conducted on 1,999 trials across 79 toddlers, as they viewed 78 unique scenes.

#### Edge extraction and characterization

We used the MATLAB Image Processing Toolbox’s edge() function to extract the edges within each image presented to participants during experimental sessions. By default, this function applies the Sobel operator (Sobel & Feldman, 1973), which computes an approximation of the gradient of the image intensity function based on changes in luminance. The output is a binary image in which each pixel is assigned a value of 1 to indicate detected edges or 0 to indicate nonedges, highlighting object boundaries and other points of luminance change. We used this edge extraction method for several reasons. First, this mirrored prior work examining the overrepresentation of cardinal edge orientations in natural scenes (Coppola et al., 1998). Second, because the Sobel method directly estimates horizontal and vertical gradients, it provides orientation information at every pixel and preserves the full distribution of edges across the image, which is important for image-level analyses. Third, the method is computationally efficient and parameter free, making it easy to apply consistently across large stimulus sets and ensuring reproducibility. Although the Canny method (Canny, 1986) is often regarded as the “gold standard” for precise edge localization and noise suppression, its thresholding and filtering steps can remove weaker edges that may meaningfully contribute to the overall distribution of orientations.

After extracting all edges, we characterized their orientations by classifying those within 22.5° of the cardinal orientations (i.e., 0°, 90°, 180°, and 270°; following the methods of Coppola et al., 1998) as cardinal edges (see Fig. 1 for examples). We quantified the *proportion of cardinal edges* by dividing the number of cardinal edges by the total number of detected edges within a scene. We defined each image’s *edge quantity* as the total number of detected edges.

We took several steps to ensure our analyses reflect infants’ and toddlers’ sensitivity to effects of edges per se (i.e., object boundaries and oriented structure) rather than other image properties that may co-occur with edges. First, we determined whether edge quantity and proportion of cardinal edges were related to other low-level image features. We used the Aesthetics Toolbox (Redies et al., 2025) to compute additional image properties including root-mean-square contrast (luminance variability), Balance (symmetry of the vertical, horizontal and diagonal image axes), mean H channel value (average hue), mean S channel value (average saturation), and mean V channel value (average brightness). Edge quantity was not significantly correlated with any of these variables (all FDR-corrected *p* values > .53) and proportion of cardinal edges was only modestly correlated to the mean S channel value (*r* = − .33, FDR-corrected *p* = .03, all other *p* values > .34). Although the proportion of cardinal edges was significantly related to saturation, this color property is theoretically distinct from the structural information carried by edges and unlikely to account for the effects we attribute to edge orientation. Nonetheless, we conducted supplementary analyses including the average saturation channel value as a covariate in the analyses of the effect of cardinal orientations (see Supplementary Materials). The patterns of results were identical to those reported here, suggesting that infants and toddlers’ engagement with scenes was not uniquely related to the overall saturation of the images.

Second, as a theoretical control, we included root-mean-square contrast (i.e., RMS contrast) as a covariate in our analyses of looking behavior. RMS contrast measures the luminance variability so this comparison allowed us to confirm that the observed effects of edge quantity specifically reflect sensitivity to edges rather than general luminance variation. Finally, in a supplemental analysis, we further isolated edge-specific effects by creating a residualized measure of edge quantity in which variance associated with RMS contrast, balance, and the mean H, S, and V channel values was statistically removed. Thus, this residualized variable provides a measure of edge quantity that is completely uncorrelated with these other low-level features. Because this residualized predictor is less directly interpretable and edge quantity was not substantially correlated with these image statistics, these analyses were treated as a robustness check. These analyses produced patterns of results highly consistent with the primary analyses, confirming that our observed effects were not driven by shared variance with other low-level image statistics (see Supplementary Materials).

### Analysis approach

Due to the differences in the testing procedures, we analyzed the data from each experiment separately. For each sample, we fit separate linear mixed-effects (LME) models on total looking time on each trial using the lme4 package (Bates et al., 2015) in R (R Core Team, 2019). We included fixed effects and all interactions between the edge structure property (proportion of cardinal orientations in the first model, edge quantity and RMS contrast in the second model) and age group. Edge quantity and RMS contrast were standardized to have mean 0 and standard deviation of 1. Age group was treated as a categorical factor that was contrast coded to aid with interpretation of effects. In the first model evaluating the role of proportion of cardinal orientations on looking time, we also included edge quantity as a covariate in the Experiment 1 (infant) model as this significantly improved model performance, χ^2^(1) = 6.12, *p* = .013. For each model, we explored whether including quadratic terms would improve the model; in most cases, models including only linear predictors had the best fit (i.e., lowest AIC values). However, in Experiment 2 (toddlers), in the first model in which we examined the effect of specific orientations including the quadratic effect of cardinal orientations improved model fit, χ^2^(2) = 10.03, *p* = .007, so we report that model here. In all models, we included random intercepts of participant to account for the repeated-measures nature of the data and of scene to account for variance due to the properties of individual scenes and to address generalizability of our effects to other stimuli (Yarkoni, 2022).

## Results

All R scripts and data included in these analyses are available online (https://osf.io/zdghu). We analyzed the data for each experiment in three stages. First, we describe infants and toddlers’ general looking behavior and task engagement. Second, we present the results of the models exploring infants’ (Experiment 1) and toddlers’ (Experiment 2) response to the proportion of cardinal edges within natural scenes. Finally, we present the results of the models exploring infants’ (Experiment 1) and toddlers’ (Experiment 2) response to the quantity of edges within natural scenes.

### Basic looking characteristics

#### Experiment 1

Basic looking characteristics are presented in Table 2. Each scene in the stimulus set was viewed by an average of 51.54 infants (*SD* = 4.50; range: 42–69). Independent-samples *t* tests revealed that in the infant sample, the older age group, on average, made more fixations per trial, *t*(105) = 3.26, *p* = .001, and looked longer overall per trial, *t*(105) = 3.54, *p* < .001, compared with the younger group. However, the older and younger infants did not significantly differ in the number of trials they completed (*p* = .98). Note that these results slightly differ from the basic looking characteristics reported in B. K. Hunter et al., (2025a, b), due to the analyses in that publication requiring different data cleaning and exclusionary criteria.

**Table 2:** Basic looking characteristics for each age group

	*N*	Average number of trials with usable data	Average number of fixations per trial	Average total looking duration per trial
Experiment 1: Infants				
5–7 months	53	38.40 (14.88)	6.82 (1.50)	2,831.23 ms (555.03)
10–12 months	54	38.46 (13.60)	7.79 (1.55)	3,234.45 ms (662.60)
Experiment 2: Toddlers				
13–15 months	38	24.68 (13.96)	10.82 (2.29)	3,389.59 ms (1,102.11)
19–21 months	41	27.17 (15.02)	11.57 (1.87)	3,510.15 ms (882.42)

*Note.* Maximum usable trials was 50 for Experiment 1 (infants) and 51 for Experiment 2 (toddlers). Maximum total looking duration was 5,000 ms for Experiment 1 (infants) and 6,000 ms for Experiment 2 (toddlers)

#### Experiment 2

Each scene was viewed by an average of 25.71 toddlers (*SD* = 4.03; range: 17–37). The younger and older toddlers did not significantly differ in the number of trials completed, the average number of fixations per trial, or the average overall looking per trial (*p* values > .12; see Table 2). These children did complete fewer trials overall compared with the infant group. This difference is not surprising given that those children were presented with these scene stimuli interleaved with trials for an unrelated task. In addition, the toddlers’ longer looking durations reflect, at least in part, the fact that they received 6-s trials with the scenes whereas the infants received 5-s trials.

### Proportion of cardinal edges

#### Experiment 1

**Fig. 2:**
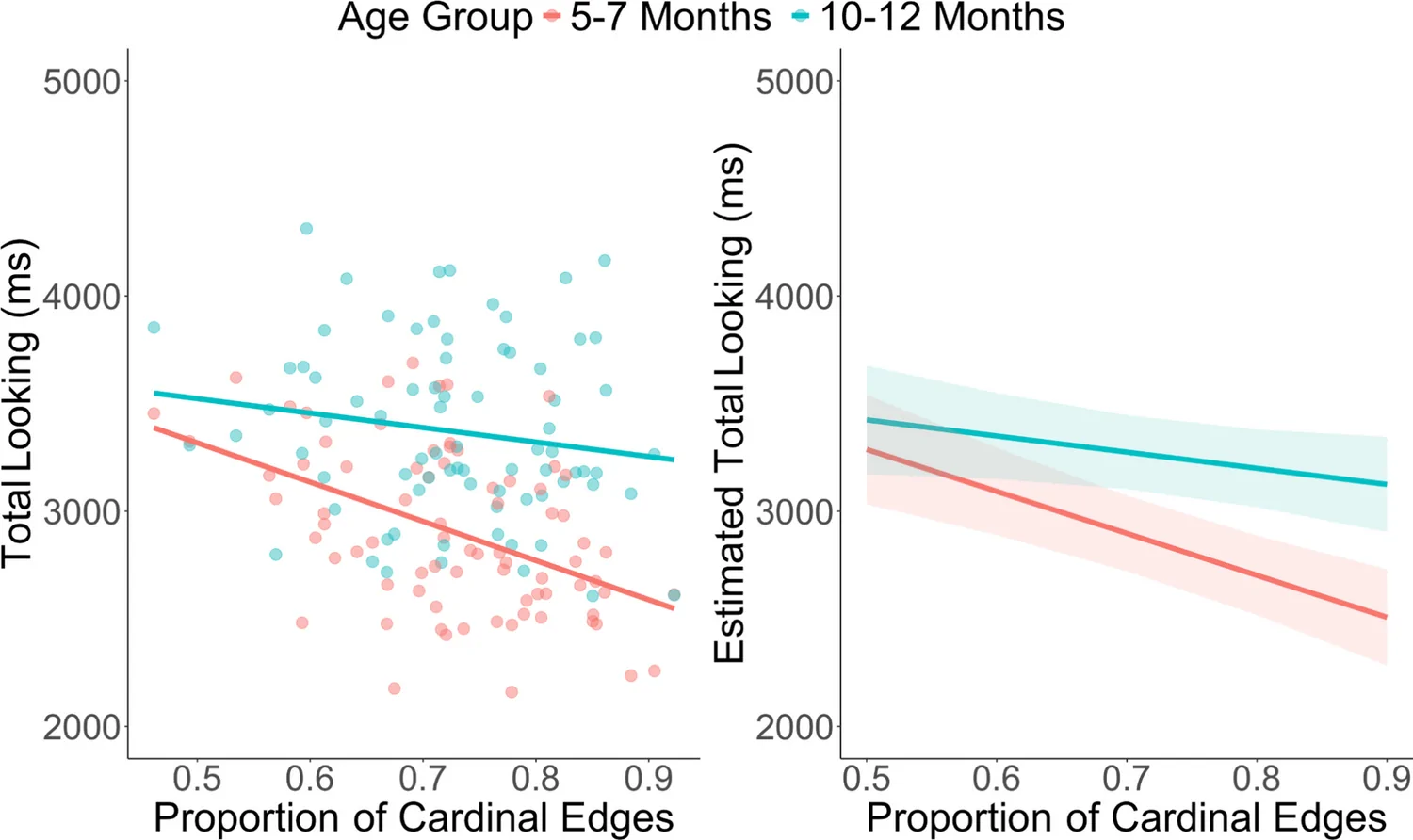
Overall looking time as a function of the proportion of edges on the cardinal axis. Left: Raw looking time by proportion of cardinal edges to the scenes. Each dot represents the average of infants’ looking time for each scene; younger infants are depicted in red and older infants are depicted in blue. The *y*-axis reflects looking time (in ms) and the *x*-axis reflects the number of cardinal edges divided by the total number of edges within a scene. Colored lines reflect best fitting linear trends in the data. Right: Estimated marginal means from the linear mixed effects model for each age group (younger infants = red; older infants = blue). The *y*-axis reflects the estimated looking time (in ms) and the *x*-axis reflects the number of cardinal edges divided by the total number of edges within a scene. Shaded regions around the lines represent 95% confidence intervals. (Color figure online)

The left panel of Fig. 2 illustrates the relation between proportion of cardinal edges and infants’ looking time. Infants of both age groups tended to look longer towards scenes with fewer cardinal edges, but this effect was stronger among the younger infants. The results of the statistical model are presented in Table 3. There were significant effects of proportion of cardinal edges and edge quantity (to be discussed in more detail in the section on Edge Quantity, below), as well as a significant interaction between age and proportion of cardinal edges. Follow-up analyses on estimated trends revealed that the strength of the relation between proportion of cardinal edges and total looking differed by age group. For younger infants, there was a significant negative effect of proportion of cardinal edges, indicating that they looked longer at scenes with proportionately fewer cardinal edges (i.e., more edges that were not near the horizontal or vertical), *t*(107) = –4.64, *p* < .001. Although the trend for the older infants was in the same direction, it was not significant, *t*(107) = − 1.81, *p* = .073, suggesting their looking was not strongly influenced by the proportion of cardinal edges. Moreover, this significant interaction between age and proportion of cardinal edges confirms that the two age groups were differently influenced by this feature of the scenes. Estimated marginal means from this model are presented in Fig. 2. As can be observed, younger and older infants did not significantly differ in their looking time to scenes with relatively few edges on the cardinal axes but compared with older infants, younger infants looked significantly less to scenes with relatively more edges on the cardinal axes.

**Table 3:** Effect of proportion cardinal edges on total looking duration, Experiment 1 (Infants)

*Predictors*	*Estimate*	*df*	*t*	*p value*
Intercept	4,031.02	81.21	14.28	< .001
Proportion of cardinal edges (linear)	− 1,350.34	75.49	− 3.58	< .001
Age group	231.13	2,068.24	1.70	.089
Edge quantity (covariate)	91.44	75.62	2.50	.014
Proportion of cardinal edges (linear) × Age group	− 599.75	3,861.64	− 3.54	< .001
**Random Effects**				
σ^2^ τ_00 participant_ τ_00 stim_ ICC *N*_participant_ *N*_stim_ Observations Marginal *R*^2^ / Conditional *R*^2^	106,732.38 308,584.88 82,856.97 0.27 107 78 4020 0.047/0.303			

*Note. *Age group was contrast-coded

#### Experiment 2

**Fig. 3:**
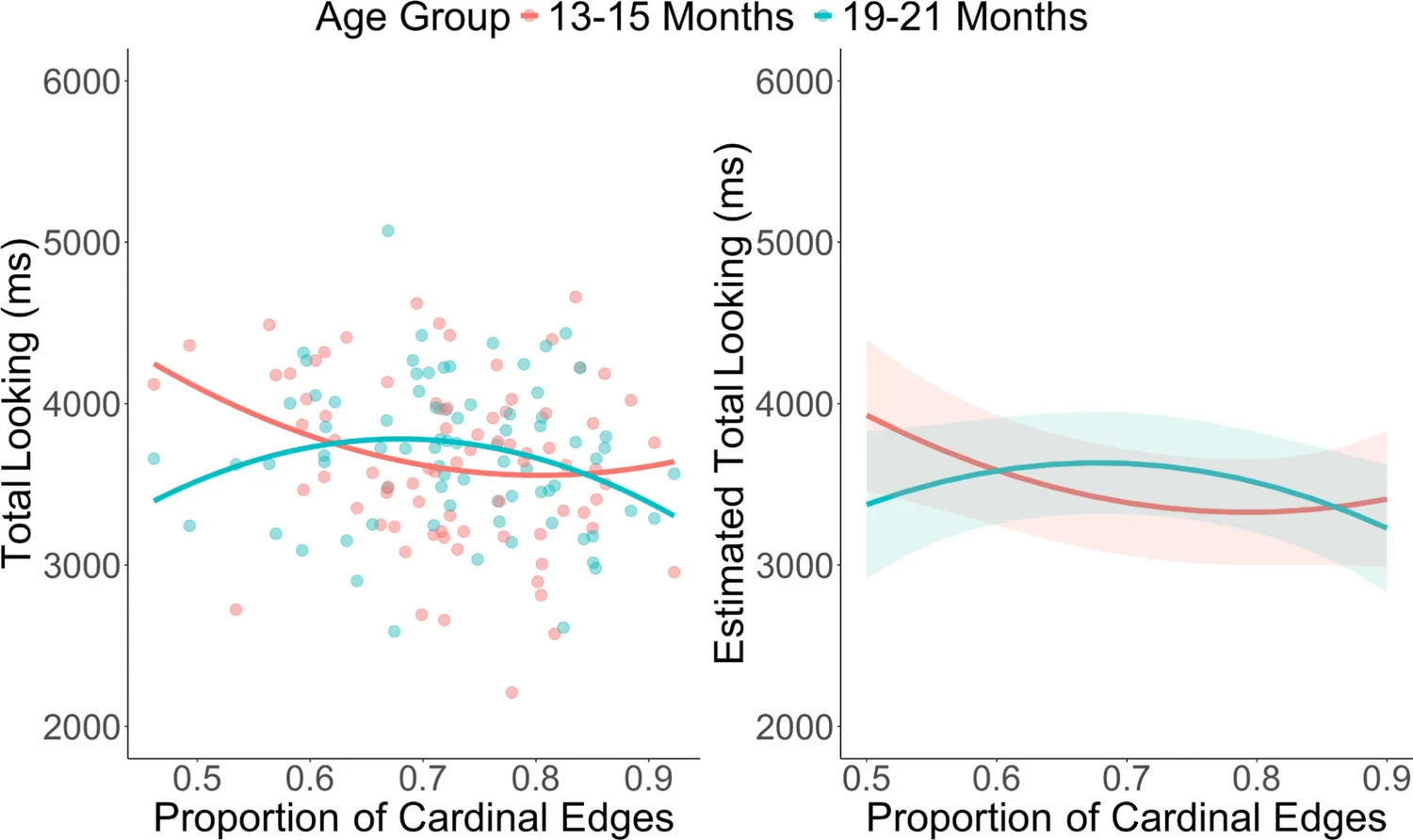
Overall looking time as a function of the proportion of edges on the cardinal axis. Left: Raw data such that each dot represents the average of toddlers’ looking time for each scene; younger toddlers are depicted in red and older toddlers are depicted in blue. The *y*-axis reflects looking time (in ms) and the *x*-axis reflects the number of cardinal edges divided by the total number of edges within a scene. Colored lines reflect best fitting linear trends in the data. Right: Estimated marginal means from the linear mixed effects model for each age group (younger toddlers = red; older toddlers = blue). The *y*-axis reflects the estimated looking time (in ms) and the *x*-axis reflects the number of cardinal edges divided by the total number of edges within a scene. Shaded regions around the lines represent 95% confidence intervals. (Color figure online)

The left panel of Fig. 3 illustrates the relation between proportion of cardinal edges and toddlers’ looking time. We found nonlinear patterns among toddler participants such that younger toddlers did not seem to vary their looking time to scenes based on the proportion of cardinal edges whereas older toddlers looked longest to scenes with a moderate proportion of cardinal edges. The results of the statistical model are presented in Table 4 and illustrated in the right panel of Fig. 3.

**Table 4:** Effect of proportion cardinal edges on total looking duration, Experiment 2 (toddler sample)

Predictors	Estimate	*df*	*t*	*p* value
Intercept	3,806.76	75.78	2.43	.017
Proportion of cardinal edges (linear)	− 22.26	74.58	− 0.01	.996
Proportion of cardinal edges (quadratic)	− 578.33	74.09	− 0.19	.853
Age group	3,939.40	1,902.18	3.28	.001
Proportion of cardinal edges (linear) × Age group	− 11,135.49	1,878.83	− 3.23	.001
Proportion of cardinal edges (quadratic) × Age group	7,620.06	1,879.12	3.15	.002
**Random effects**				
σ^2^ τ_00 participant_ τ_00 stim_ ICC *N*_participant_ *N*_stim_ Observations Marginal *R*^2^ / Conditional *R*^2^	1,583,133.51 845,457.35 42,539.29 0.36 79 78 1999 0.008/ 0.364			

*Note.* Age group was contrast coded

We observed a significant fixed effect of age, which interacted with both the linear and quadratic effects of proportion of cardinal edges. To probe these interactions, we ran additional models separately for the older and younger toddlers. For the younger toddlers, neither the linear nor the quadratic effect was significant, suggesting no clear relation between the proportion of cardinal edges and looking in these younger toddlers. For the older toddlers, in contrast, the linear effect was marginally significantly positive t(75.79) = 1.99, *p* = .050, whereas the quadratic effect was significantly negative t(74.75) = −2.09, *p* = .040, indicating an inverse U-shaped relationship between the proportion of cardinal edges and looking time. That is, for these older toddlers, there appeared to be a “sweet spot” in the middle of the distribution of the proportion of cardinal edges that resulted in longer looking, with larger and smaller proportions of cardinal edges eliciting less looking. We will return to this point in the Discussion.

### Edge quantity

#### Experiment 1

**Fig. 4:**
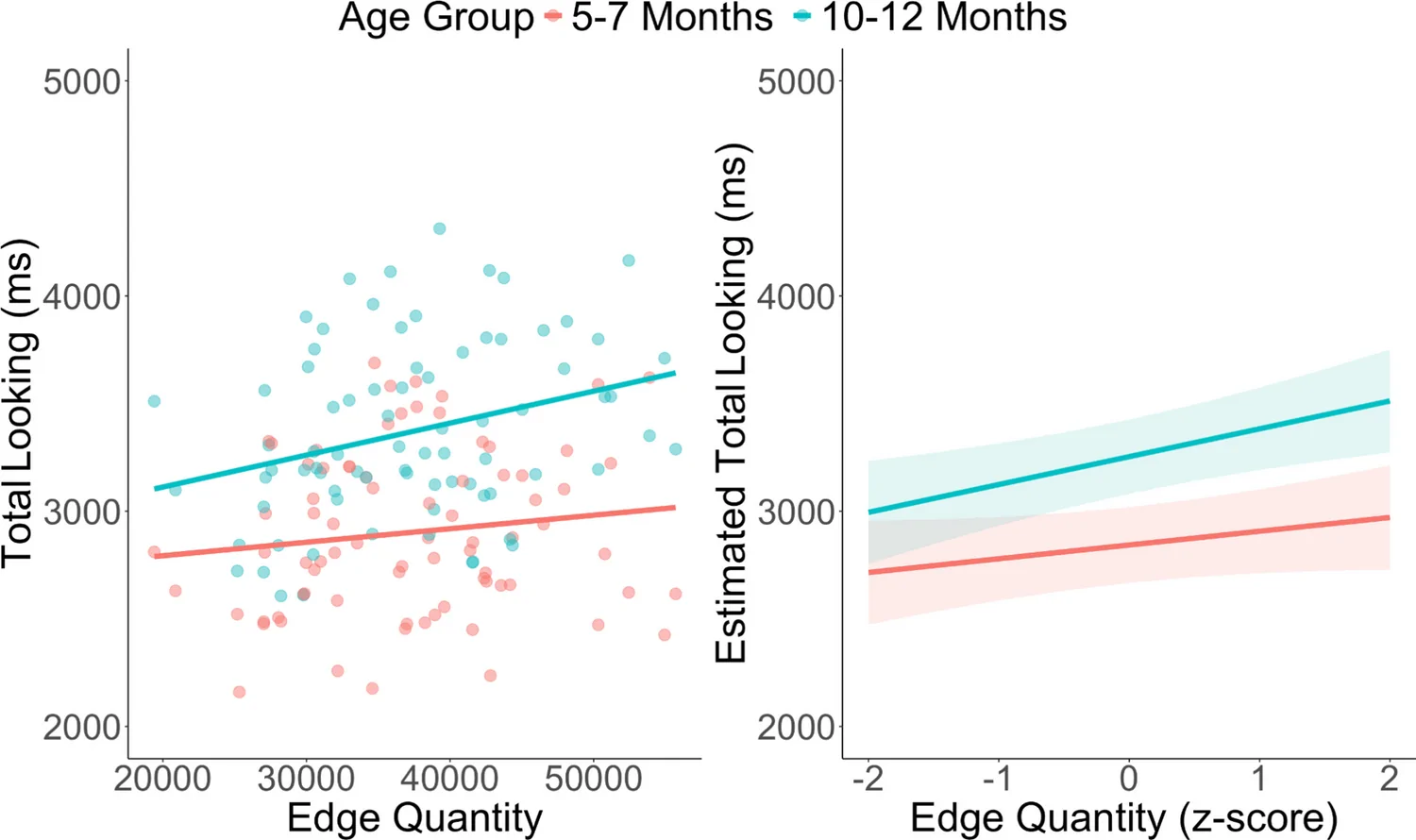
Infants’ overall looking time as a function of edge quantity. Left: Raw looking times to the scenes as a function of edge quantity. Each dot represents the infants’ average looking time for a single scene; younger infants are depicted in red and older infants are depicted in blue. The *y*-axis reflects looking time (in ms) and the *x*-axis reflects the number of edges in the scene. Colored lines reflect best fitting linear trends in the data. Right: Estimated marginal means of looking times from the linear mixed effects model for each age group (younger infants = red; older infants = blue). The *y*-axis reflects the estimated looking time (in ms) and the *x*-axis reflects the scaled edge quantity score. Shaded regions around the lines represent 95% confidence intervals. (Color figure online)

The left panel of Fig. 4 illustrates the relation between edge quantity and infants’ looking time. As can be observed, older infants looked longer towards the scenes overall compared with younger infants (i.e., the blue line is higher than the red line). In addition, older infants tended to look longer towards scenes with more edges, whereas younger infants did not show this effect.

The results of the statistical model on edge quantity are presented in Table 5. We observed significant fixed effects of age and edge quantity, as well as a significant interaction between these two fixed effects. Follow-up analyses using estimated trends revealed that the relation between edge quantity and total looking differed by age group. For older infants, there was a significant positive effect of edge quantity, indicating that they looked longer at scenes with more edges, *t*(104) = 3.09, *p* = .003. In contrast, younger infants did not show a significant effect of edge quantity, *t*(105) = 1.51, *p* = .134.

**Table 5:** Effect of edge quantity on total looking duration

	Experiment 1: Infants				Experiment 2: Toddlers			
Predictors	Estimate	*df*	*t*	*p* value	Estimate	*df*	*t*	*p* value
Intercept	3,048.39	148.62	45.62	< .001	3,482.07	78.70	31.16	< .001
Edge quantity	96.64	75.45	2.50	.015	0.52	69.68	0.01	.989
RMS contrast	77.11	75.49	1.99	.050	46.15	68.80	1.22	.226
Age group	− 205.23	101.45	− 3.60	< .001	− 55.16	72.23	− 0.51	.614
Edge quantity × Age group	− 32.71	3,859.59	− 1.98	.048	− 54.54	1,883.19	− 1.92	.055
**Random Effects**								
σ^2^ τ_00 participant_ τ_00 stim_ ICC *N*_participant_ *N*_stim_ Observations Marginal *R*^2^ / Conditional *R*^2^	1,067,076.38 308,698.81 94,479.83 0.27 107 78 4020 0.038/ 0.302				1,588,468.96 836,258.69 48,040.66 0.36 79 78 1999 0.003/ 0.360			

*Note.* For both models, age group was contrast coded

Estimated marginal means from this model are presented in the right panel of Fig. 4. As can be observed, younger and older infants did not significantly differ in their looking time to scenes with relatively few edges (lower *z* scores), but older infants looked longer than younger infants to scenes with relatively more edges (higher *z* scores). Importantly, we did not find a significant effect of RMS contrast (see Supplementary Materials for figure), indicating this edge quantity effect cannot be attributed to overall luminance variability.

#### Experiment 2

**Fig. 5:**
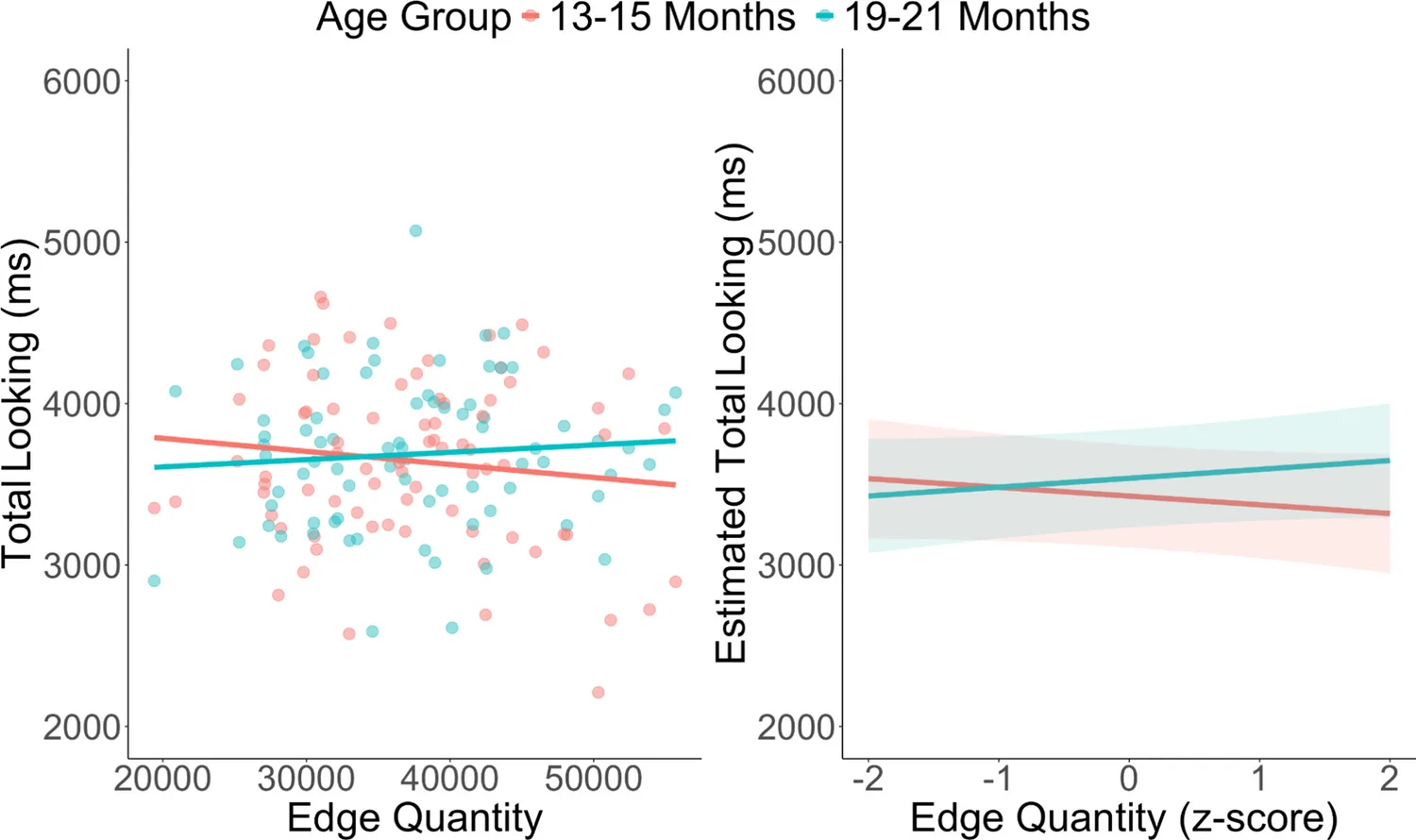
Toddlers’ overall looking time as a function of edge quantity. Left: Raw looking times to the scenes as a function of edge quantity. Each dot represents the toddlers’ average looking time for a single scene; younger toddlers are depicted in red and older toddlers are depicted in blue. The *y*-axis reflects looking time (in ms) and the *x*-axis reflects the number of edges in the scene. Colored lines reflect best fitting linear trends in the data. Right: Estimated marginal means from the linear mixed effects model for each age group (younger toddlers = red; older toddlers = blue). The *y*-axis reflects the estimated looking time (in ms) and the *x*-axis reflects the scaled edge quantity score. Shaded regions around the lines represent 95% confidence intervals. (Color figure online)

Figure 5 illustrates the relation between edge quantity and looking time for the toddler sample. It is immediately clear that the effects of edge quantity are qualitatively different for toddlers compared with what was observed with infants. Younger and older toddlers had similar overall looking times (i.e., the blue and red lines overlap) and toddlers did not appear to modulate their looking to the scenes as a function of edge quantity. The results of the statistical model presented in Table 5 confirm this impression. It did not yield any statistically significant main effects or interactions with age or edge quantity or RMS contrast (see Supplementary Materials for RMS figure).

## Discussion

This study examined the influence of edges and edge orientations on both infant and toddler attentional engagement toward natural scenes. We found developmental changes in the influence of both the orientation and quantity of edges on infants’ attention, indicating that the low-level features that sustain attention do not exert uniform effects across early development. Younger but not older infants had significantly shorter looking durations as the proportion of cardinal edges increased. At the same time, older but not younger infants’ looking duration increased as the quantity of edges increased. Thus, there were age-related changes in how the edge structure of the scenes influenced infant attentional engagement.

The results for the toddler sample differed. There were nonlinear effects of the proportion of cardinal edge orientations on toddler looking time, such that older toddlers had a “sweet spot” in the middle of the distribution of the proportion of cardinal edges that elicited the most visual engagement. In contrast, toddlers’ attention appeared to be less influenced by the quantity of edges in the scenes. Thus, only the effect of proportion of cardinal edges on toddler looking changed across development. Importantly, at this age, sensitivity to edges did not follow a simple linear trajectory.

We found that attention by both infants and toddlers was systematically related to the proportion of cardinal orientations within scenes. Prior work demonstrates that adults’ and infants’ visual inputs show an overrepresentation of cardinally oriented edges (Coppola et al., 1998; Petroff et al., 2025) and that experience with specific orientations in the environment may have cascading influences on adults’ perceptual skills (Blasdel et al., 1977). In our sample, infants attended longer to scenes with relatively fewer cardinal orientations. This pattern is consistent with their looking times being driven by preference for scenes that are relatively novel in edge structure given their typical visual experiences (i.e., their visual diets). It is well established that infants rapidly learn environmental language regularities, such as commonly occurring syllables in natural speech patterns, through statistical learning mechanisms (see Saffran & Kirkham, 2018). Newman et al. (2025) proposed that these same statistical learning mechanisms may facilitate infants’ learning of the visual properties in the natural world, directly influencing perceptual sensitivities to different visual features.

Sensitivity to frequently experienced features may shift across development as the visual environment interacts with other changes, such as developing postural control (Luo & Franchak, 2020) or the transition from crawling to walking (Kretch et al., 2014). Specifically, the edge structure of infants’ visual diet is constrained by such postural and motor abilities and thus across development infants experience different levels of edge quantity and proportions of cardinal edges. We found changes in the role of edge structure consistent with this perspective. Specifically, the role of the proportion of cardinal orientations on visual attention was weaker among older infants compared with younger. As infants approach their first birthday, they spend a larger proportion of their time in an upright position as they learn to stand and walk (Franchak, 2019). Their position and size of objects in their visual field changes as they become more adept at locomoting toward, reaching for, and manipulating objects (Smith et al., 2011). These changes may increase the proportion of cardinal edges in infants’ egocentric views of the world, and as a result the variation in cardinal edges in our stimuli was less compelling.

The older toddlers demonstrated an inverse-U-shaped pattern, indicating that attention may be sustained the longest when the proportion of cardinal orientations falls within an ideal range based on the perceptual and cognitive abilities of the child. This interpretation is consistent with the notion of a “Goldilocks effect” such that the attention system prioritizes information that is not too redundant nor too complex (Kidd et al., 2012). The fact that this pattern was observed for the older but not younger toddler sample suggests that there are developmental shifts in the second year such that the optimal proportion of cardinal edges changes. Importantly, this shift is not likely to be due to postural and motor changes but rather likely reflects development in other domains. In general, our findings are consistent with the hypotheses proposed by Newman et al. (2025), and further suggest that visual statistical learning of the natural world may not only influence perceptual sensitivities but also attentional sensitivities.

An alternative possibility is that these effects instead reflect the developing structural organization of the visual system. The horizontal and vertical meridians serve as fundamental boundaries for the topographic mapping of visual fields in early visual cortex (Essen & Zeki, 1978; Gattass et al., 1981), which may guide attention independently of experience. Because environmental statistics and cortical architecture are confounded in typical development, it is unclear whether our observed effects reflect a sensitivity to visual diet, experience-independent neural structure, or an experience-dependent tuning of underlying structures. Future research relating individual differences in visual diets to attentional patterns will be necessary to disentangle these competing influences. Nonetheless, our results indicate dynamic changes in how orientation-based information is weighted in the attentional system across the first two postnatal years of life.

We observed that only the older infants showed an effect of edge quantity on their looking time; they differed significantly from the younger infants. No differences were observed for the toddler sample. Because prior work suggests that edge density is highly correlated with adults’ subjective ratings of visual complexity (Rosenholtz et al., 2007), our findings suggest that the older infants may prefer scenes that are more complex or information rich. This conclusion is consistent with that from prior work examining infants’ attention to relatively simple stimuli (e.g., checkerboards). In these now classic studies, infants look longer to more “complex” displays, or displays with more information (e.g., more checks; Cohen et al., 1975). Although edge quantity was not significantly related to younger infants’ looking time, this does not mean that these infants do not also prefer complex visual stimuli. Several studies have shown that although infants in general prefer more complex stimuli, the highest level of complexity preferred increases with age (Brennan et al., 1966; Hershenson, 1964). It is possible that the distribution of “complexity,” or edge quantity, in our stimuli did not represent a range that could capture these younger infants’ preference for more complex stimuli. An alternative possibility is that immaturity in the younger infants’ visual acuity may have made some edges in our scenes less perceptible. Future work may explore these possibilities by varying edge detection methods to reduce noise (e.g., adjusting kernel sizes), blurring images pre-edge extraction, or more systematically varying the edge quantity in the scenes to increase the range.

Likewise, it may also be incorrect to conclude that the lack of significant effects among toddlers suggests that their attention is unrelated to the complexity of the scenes. Just as the range of edge densities may not have captured the preference for complexity in our youngest infants, it also may not have captured the preference for complexity in our toddlers. Moreover, “complexity” is subjectively defined differently across development, with complexity for infants being reflected in low-level visual properties whereas complexity for toddlers being increasingly reflected in higher-order semantic concepts. Alternatively, task differences across the two experiments may have contributed to the null findings for edge quantity among toddlers.

Including toddlers in our study allowed for significant understanding of development overall, given the relative lack of research with toddlers in the attention literature (Kerr-German et al., 2023). Although this period of development is marked by dramatic changes in motor and language development (Açık et al., 2010; Helo et al., 2017), relatively less is known about which specific features of the environment guide where and for how long toddlers look. Our work contributes to filling this gap by identifying that specific orientations are important for sustaining toddlers’ visual attention. Our nonlinear effects that varied across the two age groups during this developmental period suggest that toddlerhood may be a time of rapid reorganization of which features of the environment are most influential for engaging attention. This period is also marked by changes in visual object recognition (Smith, 2009), dorsal stream functioning for object rotation and processing of spatiotemporal events (Street et al., 2011), as well as prefrontal cortex (Diamond, 2002). It is possible that these co-occurring developments interact with each other, dynamically shifting the visual features of the environments that are attended to. These patterns underscore the importance of modeling developmental change flexibly, rather than assuming monotonic increases or decreases in feature sensitivity.

The relatively small effect sizes in the present study underscore that low-level edge structure is only one of many factors that determine visual attention. Future work should investigate other image statistics and explore how sensitivity to low-level features interacts with attention to higher-order features, such as semantic meaning, familiarity, and social relevance. For example, infants and toddlers may heavily rely on edge structure in unfamiliar environments, where low-level structure provides cues for organizing visual input, but the role of these features may decrease in contexts where the child can rely on learned semantic associations. In the present study, we intentionally selected a stimulus set that should be generally familiar to all participants; varying semantic categories of scenes and assessing individual differences in content familiarity may address this possibility. Future work should also consider how constraints in motor ability relate to edge sensitivities. Evolving motor abilities (e.g., the ability to self-locomote) increasingly allow infants to actively control their visual inputs (Corbetta & Snapp-Childs, 2009; Smith et al., 2018), and these abilities may modulate the features that are prioritized by the attention system. Relatedly, infants’ increasing ability to control their head and maintain an upright head posture affects which real-world edges are indeed viewed as cardinal (e.g., a 45° edge becomes cardinal when the head is tilted). Finally, examining attention to features of dynamic stimuli (i.e., videos) may reveal different patterns than the static images presented in this study. A child’s visual experience of the real world comes in the form of dynamic information rather than distinct, static frames. Understanding how attention systems weigh low-level information such as edges and their orientations in these contexts will be essential for building comprehensive models of developing attentional systems.

Overall, our findings suggest that early attention is sensitive to both the orientation and quantity of edges, but that the relative importance of these features changes with development. Infants’ looking durations were significantly related to both edge properties of scenes; they looked less to scenes with a higher proportion of cardinal edges and longer to scenes with more edges. Toddlers’ looking was not related to the number of edges, but their looking durations had a nonlinear relation with the proportion of cardinal edges in scenes. Together, these results demonstrate that the weight of different low-level visual features in attention engagement is not static but rather reorganizes substantially within infancy and toddlerhood, offering new insights into how the visual system prioritizes information during early development.

## Supplementary Information

Below is the link to the electronic supplementary material.

Supplementary file1 (DOCX 2904 kb)

## Data Availability

The data that support the findings of this study and full stimulus set are openly available on OSF (https://osf.io/zdghu).

## Footnotes

1 Welch’s *t* tests revealed no significant differences in edge structure between indoor and outdoor scenes or between scenes containing people/animals and those without biologically relevant content (all *p* values > .05), indicating that these semantic categories were not systematically associated with the edge metrics examined here.

2 Because the toddlers in Experiment 2 always looked at the attention getter before the trial began, our primary results reported here exclude trials in Experiment 1 in which the infant did not look at the attention getter before the trial. Thus, across both experiments, all included trials began with participants engaged. We also conducted secondary analyses using revised exclusionary criteria, including the manually progressed trials in which the infant made more than one fixation on the scene during the 5-s trial. The results of these secondary analyses are nearly identical to those reported here and are reported in the Supplementary Materials.

## Code availability

The analysis script that support the findings of this study is openly available on OSF (https://osf.io/zdghu).
