# Supplementary material for: Edge structure influences infants’ and toddlers’ engagement with natural scenes
Source: Atten Percept Psychophys. 2026 Oct 28;88(8):212. doi: 10.3758/s13414-026-03357-x (PMC13719721; doi:10.3758/s13414-026-03357-x)
Supplement: Supplementary file 1 [file 13414_2026_3357_MOESM1_ESM.docx]

Supplementary Materials for

**Edge Structure Influences Infants’ and Toddler’s Engagement with Natural Scenes**

Brianna K. Hunter, Kaitlyn Wong, & Lisa M. Oakes

**Contents**

S1. Restricted Analyses of Experiment 2 Data

S2. Expanded Analyses of Experiment 1 Data

S3. Controlling for Average Saturation Channel Values

S4. Residualized Measure of Edge Quantity

S5. Supplementary Figures of RMS Contrast Results

**S1. Restricted Analyses of Experiment 2 Data**

Seven of these toddlers in Experiment 2 were also a part of the 5-7 month infant sample in Experiment 1. Although the sessions were spaced far apart (*M* = 10.766 months, *SD* = 3.48, range: 7.14 - 15.65 months), the overall testing procedures differed, and only a subset of scenes were randomly selected from a larger pool during each visit, we conducted supplemental analyses removing the scenes from the toddler sample (i.e., Experiment 2) that had been previously viewed.

**Edge Quantity**

As observed in Table S1, the results were consistent when removing the trials viewed for a second time during a second visit to the lab. That is, we did not observe any statistically significant main effects relating to age or edge quantity. The only difference in the results was that the marginally significant interaction between these two fixed effects observed in the primary analyses became significant in these analyses. However, post hoc analyses of estimated trends again revealed that the direction of the slopes differed by age group, but slopes were not significantly different than zero for either the older toddlers, *t*(164) = 1.32, *p* = .189, or the younger toddlers, *t*(192) = -1.21, *p* = .228. Thus, the conclusions drawn in the main text do not vary here.

***Table S1***

*Effect of Edge Quantity on Toddler’s Total Looking Duration*

|  | **Primary Analysis Experiment 2** | | | |  | **Restricted Analysis Experiment 2** | | | |
| --- | --- | --- | --- | --- | --- | --- | --- | --- | --- |
| Predictors | Estimate | df | t | p-value |  | Estimate | df | t | p-value |
| Intercept | 3482.07 | 78.70 | 31.16 | < .001 |  | 3489.29 | 77.73 | 31.47 | < .001 |
| Edge Quantity | 0.52 | 69.68 | 0.01 | .989 |  | 1.35 | 67.86 | 0.07 | .971 |
| RMS Contrast | 46.15 | 68.80 | 1.22 | .226 |  | 43.15 | 68.64 | 1.18 | .244 |
| Age Group | -55.16 | 72.23 | -0.51 | .614 |  | -69.23 | 72.20 | -0.66 | .535 |
| Edge Quantity x Age Group | -54.54 | 1883.19 | -1.92 | .055 |  | -59.19 | 71.94 | 0.09 | .039 |
| **Random Effects**  σ^2^  τ_00_ _participant_  τ_00_ _stim_  ICC  N _participant_  N _stim_  Observations  Marginal R^2^ / Conditional R^2^ | 1067076.38  308698.81  94479.83  0.27  107  78  4020  0.038/  0.302 |  |  |  |  | 1575770.93  823087.65  40545.01  0.35  79  78  1928  0.004/  0.357 |  |  |  |

*Note.* Age group was contrast-coded

**Proportion of Cardinal Edges**

As observed in Table S2, the results regarding the role of proportion of cardinal edges were nearly identical when removing the trials viewed for a second time during a second visit to the lab. We again observed a significant fixed effect of age, which interacted with both the linear and quadratic effects of proportion of cardinal edges. Additional models run separately for the older and younger toddlers revealed that the direction of the linear and quadratic effects were opposite for older vs. younger toddlers. These follow-up analyses mirror the results reported in the main text. For the younger toddlers, the linear effect was marginally significantly negative, t(69.47) = -1.99, p = .050, whereas the quadratic effect was nonsignificantly positive, t(68.91) = 1.79, p = .078. For the older toddlers, the linear effect was marginally significantly positive t(73.38) = 2.34, p = .022, whereas the quadratic effect was significantly negative t(72.42) = -2.46, p = .016, indicating an inverse U-shaped relationship between the proportion of cardinal edges and looking time.

**Table S2**

*Effect of Proportion Cardinal Edges on Total Looking Duration*

|  | **Primary Analysis Experiment 2** | | | |  | **Restricted Analysis Experiment 2** | | | |
| --- | --- | --- | --- | --- | --- | --- | --- | --- | --- |
| Predictors | Estimate | df | t | p-value |  | Estimate | df | t | p-value |
| Intercept | 3806.76 | 75.78 | 2.43 | .017 |  | 3708.07 | 75.43 | 2.46 | .016 |
| Proportion of Cardinal Edges (linear) | -22.26 | 74.58 | -0.01 | .996 |  | 380.73 | 74.06 | 0.09 | .929 |
| Proportion of Cardinal Edges (quadratic) | -578.33 | 74.09 | -0.19 | .853 |  | -924.66 | 73.44 | -0.31 | .758 |
| Age Group | 3939.40 | 1902.18 | 3.28 | .001 |  | 4281.04 | 1833.38 | 3.45 | .001 |
| Proportion of Cardinal Edges (linear) x Age Group | -11135.49 | 1878.83 | -3.23 | .001 |  | -12101.16 | 1812.30 | -3.46 | .001 |
| Proportion of Cardinal Edges (quadratic) x Age Group | 7620.06 | 1879.12 | 3.15 | .002 |  | 8264.32 | 1813.04 | 3.37 | < .001 |
| Random Effects  σ^2^  τ_00_ _participant_  τ_00_ _stim_  ICC  N _participant_  N _stim_  Observations  Marginal R^2^ / Conditional R^2^ | 1583133.51  845457.35  42539.29  0.36  79  78  1999  0.008/0.364 | |  |  |  | 1569534.08  834704.01  32873.51  0.36  79  78  1928  0.010/0.362 | |  |  |

*Note.* Age group was contrast-coded.

**S2. Expanded Analyses of Experiment 1 Data**

In the primary analyses of Experiment 1 data, we excluded any trial in which the experimenter manually progressed the trial (i.e., the infant did not automatically trigger the trial presentation by looking at the attention getter). This exclusionary criterion was implemented to mirror the data in Experiment 2, in which all toddlers were engaged with the task at the beginning of the trial (i.e., the toddlers always automatically triggered the trial presentation by looking at the attention getter). Manual progression of trials in Experiment 1 was relatively rare (5.66% of all trials) and typically occurred when the infant was generally uninterested in the task or fussy. However, in some cases, the presentation of the scene resulted in the infants’ re-engaging with the task and contributing usable data on these trials. In these expanded analyses, we include data from these trials in which the infants re-engaged. Rather than 4020 trials across 107 infants in the main text, these analyses reflect 4256 trials across 108 infants.

**Edge Quantity**

As observed in Table S3, the results were similar when adjusting the exclusionary criteria. We again observed significant fixed effects of age and edge quantity. The interaction between these two effects was significant in the primary analyses but only marginally significant in these expanded analyses. Follow-up analyses using estimated trends again revealed that the relationship between edge quantity and total looking differed by age group. Older infants looked longer at scenes with more edges (*p* = .003), whereas younger infants did not show a significant effect of edge quantity (*p* = .134).

**Table S3**

*Effect of Edge Quantity on Total Looking Duration, Experiment 1 (Infants)*

|  | **Primary Analysis Experiment 1** | | | |  | **Expanded Analysis Experiment 1** | | | |
| --- | --- | --- | --- | --- | --- | --- | --- | --- | --- |
| Predictors | Estimate | df | t | p-value |  | Estimate | df | t | p-value |
| Intercept | 3048.39 | 148.62 | 45.62 | < .001 |  | 3153.56 | 141.79 | 43.79 | < .001 |
| Edge Quantity | 96.64 | 75.45 | 2.50 | .015 |  | 93.92 | 74.18 | 2.48 | .015 |
| RMS Contrast | 77.11 | 75.49 | 1.99 | .050 |  | 78.32 | 73.96 | 2.07 | .042 |
| Age Group | -205.23 | 101.45 | -3.60 | < .001 |  | -355.27 | 103.02 | -5.70 | < .001 |
| Edge Quantity x Age Group | -32.71 | 3859.59 | -1.98 | .048 |  | -31.24 | 73.72 | -1.78 | .076 |
| **Random Effects** | | |  |  |  |  | | |  |
| σ^2^  τ_00_ _participant_  τ_00_ _stim_  ICC  N _participant_  N _stim_  Observations  Marginal R^2^ / Conditional R^2^ | 1067076.38  308698.81  94479.83  0.27  107  78  4020  0.038/  0.302 | |  |  |  | 1278297.32  399128.49  86498.75  0.28  108  78  4256  0.074/  0.329 | | |  |

*Note.* For both models, age group was contrast-coded.

**Proportion of Cardinal Edges**

As observed in Table S4, the results were nearly identical when adjusting the exclusionary criteria. There were significant effects of proportion of cardinal edges and edge quantity (confirming the results presented above), as well as a significant interaction between age and proportion of cardinal edges. Follow-up analyses on estimated trends again revealed that the strength of the relationship between proportion of cardinal edges and total looking differed by age group. Younger infants looked longer at scenes with proportionately fewer cardinal edges (*p* < .001). In the primary analyses, there was a trend-level relation between older infants’ looking time and proportion of cardinal edges in the scenes (*p* = .073); this effect became significant with this revised exclusionary criterion (p = .048). The primary conclusions drawn in the main text do not change: the effect of cardinal edges was stronger among the younger infants.

**Table S4**

*Effect of Proportion Cardinal Edges on Total Looking Duration, Experiment 1 (Infants)*

|  | **Primary Analysis Experiment 1** | | | |  | **Expanded Analysis Experiment 1** | | | |
| --- | --- | --- | --- | --- | --- | --- | --- | --- | --- |
| Predictors | Estimate | df | t | p-value |  | Estimate | df | t | p-value |
| Intercept | 4031.02 | 81.21 | 14.28 | < .001 |  | 4155.75 | 82.69 | 15.10 | < .001 |
| Proportion of Cardinal Edges (linear) | -1350.34 | 75.49 | -3.58 | < .001 |  | -1380.26 | 75.04 | -3.78 | < .001 |
| Age Group | 231.13 | 2068.24 | 1.70 | .089 |  | 49.19 | 1924.41 | 0.34 | .735 |
| Edge Quantity (covariate) | 91.44 | 75.62 | 2.50 | .014 |  | 87.98 | 75.11 | -2.48 | .015 |
| Proportion of Cardinal Edges (linear) x Age Group | -599.75 | 386.64 | -3.54 | < .001 |  | -556.21 | 4093.67 | -3.09 | .002 |
| **Random Effects** | | |  |  |  |  | | |  |
| σ^2^  τ_00_ _participant_  τ_00_ _stim_  ICC  N _participant_  N _stim_  Observations  Marginal R^2^ / Conditional R^2^ | 106732.38  308584.88  82856.97  0.27  107  78  4020  0.047/  0.303 | |  |  |  | 1276358.11  399203.98  73801.58  0.27  108  78  4256  0.082/  0.330 | | |  |

*Note.* For both models, age group was contrast-coded.

**S3. Controlling for Average Saturation Channel Value of Scenes**

As described in the main text, our goal was to ensure that analyses reflected infants’ and toddlers’ sensitivity to effects of edges rather than other image properties that may co-occur with edges. The average saturation value of the images was modestly correlated with the proportion of cardinal edges within the scenes (*r* = -.33). We therefore conducted these supplementary analyses on the effects of cardinal orientations, including the average saturation channel value as a covariate in the analyses. When including the average saturation channel value as a covariate in the statistical analyses, the overall pattern of results for both infants (Table S3) and toddlers (Table S4) was nearly identical to those reported in the main text.

***Table S3***

*Effect of Cardinal Orientations on Total Looking Duration: Experiment 1*

|  | **Primary Analysis** | | | |  | **Supplemental Analysis** | | | |
| --- | --- | --- | --- | --- | --- | --- | --- | --- | --- |
| *Predictors* | *Estimate* | *df* | *t* | *p-value* |  | *Estimate* | *df* | *t* | *p-value* |
| Intercept | 4031.02 | 81.21 | 14.28 | < .001 |  | 3984.67 | 79.55 | 13.30 | < .001 |
| Proportion of Cardinal Edges (linear) | -1350.34 | 75.49 | -3.58 | < .001 |  | -1286.58 | 74.56 | -3.20 | .002 |
| Age Group | 231.13 | 2068.24 | 1.70 | .089 |  | 231.01 | 2067.80 | 1.70 | .089 |
| Edge Quantity (covariate) | 91.44 | 75.62 | 2.50 | .014 |  | 92.76 | 74.61 | 2.52 | .014 |
| Mean Saturation Channel (covariate) | NA | NA | NA | NA |  | 18.56 | 75.28 | 0.48 | .633 |
| Proportion of Cardinal Edges (linear) x Age Group | -599.75 | 3861.64 | -3.54 | < .001 |  | -599.72 | 3861.49 | -3.54 | < .001 |
| **Random Effects**  σ^2^  τ_00_ _participant_  τ_00_ _stim_  ICC  N _participant_  N _stim_  Observations  Marginal R^2^ / Conditional R^2^ | 106732.38  308584.88  82856.97  0.27  107  78  4020  0.047/0.303 | |  |  |  | 1064733.16  308647.39  83904.72  0.27  107  78  4020  0.047/0.304 |  |  |  |

*Note.* For both models, age group was contrast-coded.

***Table S4***

*Effect of Cardinal Orientations on Total Looking Duration: Experiment 2*

|  | **Primary Analysis** | | | |  | **Supplemental Analysis** | | | |
| --- | --- | --- | --- | --- | --- | --- | --- | --- | --- |
| *Predictors* | *Estimate* | *df* | *t* | *p-value* |  | *Estimate* | *df* | *t* | *p-value* |
| Intercept | 3806.76 | 75.78 | 2.43 | .017 |  | 3495.24 | 74.82 | 2.21 | .030 |
| Proportion of Cardinal Edges (linear) | -22.26 | 74.58 | -0.01 | .996 |  | 701.52 | 73.67 | 0.16 | .875 |
| Proportion of Cardinal Edges (quadratic) | -578.33 | 74.09 | -0.19 | .853 |  | -976.09 | 73.22 | -0.31 | .755 |
| Age Group | 3939.40 | 1902.18 | 3.28 | .001 |  | 3936.29 | 1902.38 | 3.26 | .001 |
| Mean Saturation Channel (covariate) |  |  |  |  |  | 47.17 | 73.35 | 1.21 | .229 |
| Proportion of Cardinal Edges (linear) x Age Group | -11135.49 | 1878.83 | -3.23 | .001 |  | -11128.30 | 1879.03 | -3.23 | .001 |
| Proportion of Cardinal Edges (quadratic) x Age Group | 7620.06 | 1879.12 | 3.15 | .002 |  | 7616.07 | 1879.32 | 3.15 | .002 |
| **Random Effects**  σ^2^  τ_00_ _participant_  τ_00_ _stim_  ICC  N _participant_  N _stim_  Observations  Marginal R^2^ / Conditional R^2^ | 1583133.51  845457.35  42539.29  0.36  79  78  1999  0.008/  0.364 | |  |  |  | 1583033.03  845616.62  42023.12  0.36  79  78  1999  0.008/0.365 |  |  |  |

*Note.* For both models, age group was contrast-coded.

**S4. Residualized Measure of Edge Quantity**

As described in the main text, our goal was to ensure that analyses reflected infants’ and toddlers’ sensitivity to effects of edges rather than other image properties that may co-occur with edges. One approach to this was creating a residualized measure of edge quantity by statistically removing variance associated with RMS contrast (luminance variability), balance (symmetry of the vertical, horizontal and diagonal image axes), and the mean H (hue), S (saturation), and V (value, brightness) channel values. This residualized variable therefore provides a measure of edge quantity that is independent of these other low-level features.

***Experiment 1 (Infants)***

When using a residualized measure of edge quantity, the overall pattern of results was similar to those reported in the main text. We observed significant fixed effects of age and edge quantity (Table S3). However, the marginally significant interaction between these two fixed effects became non-significant, suggesting that infants across the first postnatal year looked longer to scenes with more edges.

***Experiment 2 (Toddlers)***

When using a residualized measure of edge quantity, the overall pattern of results was again similar to those reported in the main text. However, the marginally significant interaction between age and edge quantity reported in the main text became significant when using the residualized edge quantity measure (see Table S3). Post hoc analyses of estimated trends again revealed that the direction of the slopes differed by age group, but slopes were not significantly different than zero for either the older toddlers, *t*(160) = 1.23, *p* = .219, or the younger toddlers, *t*(183) = -1.38, *p* = .171.

**Table S5**

*Effect of Edge Quantity (residualized) on Total Looking Duration*

|  | **Experiment 1: Infants** | | | |  | **Experiment 2: Toddlers** | | | |
| --- | --- | --- | --- | --- | --- | --- | --- | --- | --- |
| Predictors | Estimate | df | t | p-value |  | Estimate | df | t | p-value |
| Intercept | 3048.52 | 148.98 | 45.54 | < .001 |  | 3482.44 | 78.66 | 31.18 | < .001 |
| Edge Quantity | 90.76 | 75.40 | 2.33 | .022 |  | -4.48 | 69.89 | -0.12 | .906 |
| RMS Contrast | 74.80 | 75.51 | 1.92 | .059 |  | 46.46 | 68.95 | 1.23 | .222 |
| Age Group | -205.22 | 101.44 | -3.60 | < .001 |  | -54.76 | 72.24 | -0.50 | .617 |
| Edge Quantity x Age Group | -24.02 | 3859.54 | -1.45 | .146 |  | -61.64 | 1882.76 | -2.18 | .030 |
| **Random Effects** | | |  |  |  |  | | |  |
| σ^2^  τ_00_ _participant_  τ_00_ _stim_  ICC  N _participant_  N _stim_  Observations  Marginal R^2^ / Conditional R^2^ | 1067558.00  308827.12  95631.57  0.27  107  78  4020  0.037/  0.301 | |  |  |  | 1587815.19  835828.58  47734.72  0.36  79  78  1999  0.004/  0.360 | | |  |

*Note.* For both models, age group was contrast-coded.

**S5. Supplementary Figures for RMS Contrast Results**

INFANTS

TODDLERS
